# Comparing the cognitive functioning of middle-aged and older foreign-origin population in Estonia to host and origin populations

**DOI:** 10.3389/fpubh.2023.1058578

**Published:** 2023-07-12

**Authors:** Liili Abuladze, Luule Sakkeus, Elena Selezneva, Oksana Sinyavskaya

**Affiliations:** ^1^Estonian Institute for Population Studies, School of Governance, Law and Society, Tallinn University, Tallinn, Estonia; ^2^Estonian Interuniversity Population Research Centre, Tallinn, Harju County, Estonia; ^3^Population Research Institute, Väestöliitto, Helsinki, Finland; ^4^Independent Researcher, Moscow, Russia

**Keywords:** aging, migrant health, cognitive functioning, immediate recall, verbal fluency

## Abstract

**Background:**

In migration and health research, the healthy migrant effect has been a common finding, but it usually pertains to specific contexts only. Existing findings are inconsistent and inconclusive regarding the cognitive functioning of the (aging) foreign-origin population relative to the populations of their host and sending countries. Moreover, this comparison is an understudied design setting.

**Objective:**

We analyze the outcomes and associations of cognitive functioning outcomes of the non-institutionalized middle-aged and older population, comparing the Russian-origin population in Estonia with Estonians in Estonia and Russians in Russia in a cross-sectional design. We aim to estimate the (long-term) effects of migration on cognitive functioning in later life, contextualizing the findings in previous research on the healthy migrant effect.

**Data and methods:**

We use data from face-to-face interviews conducted within the SHARE Estonia (2010–2011) and SAGE Russia (2007–2010) surveys. Respondents aged 50+ living in urban areas were grouped by self-identified ethnicity, including 2,365 Estonians, 1,373 Russians in Estonia, and 2,339 Russians in Russia (total *N* = 6,077). Cognitive functioning was measured using a 25-percentile cut-off threshold for the results of two cognition outcomes - immediate recall and verbal fluency - and the odds of impairment were estimated using binary logistic regression.

**Results:**

Russian men and women living in Estonia have significantly higher odds of impairment in immediate recall than Estonian men and women, though they do not differ from Russians in Russia in the final adjusted models. The differences between all groups are non-significant if age at migration is considered. There are no significant differences between the groups in verbal fluency.

**Conclusion:**

Contrary to the commonly found healthy migrant effect, the middle-aged and older foreign-origin population in Estonia fares initially worse than the native population in the immediate recall outcome, but does not differ from their sending country population, possibly due to Russia’s higher mortality rate and therefore the selective survival of healthier people. Different results depending on the cognitive functioning outcome suggest that migration may affect temporary memory more than crystallized knowledge. However, there are no differences between the groups if defined based on age at migration, which suggests that the age profile differences explain most of the groups’ differences in cognitive functioning.

## 1. Introduction

Studies on health and migration have consistently found support for the healthy migrant effect, which contributed to improving the population-level health and mortality outcomes [e.g., (1–3)]. However, more mixed results can be found when the origin and length of the residence of migrants is considered [e.g., (4–6)]. Most of the literature has focused on Western countries and on younger migrants, though exceptions in Eastern Europe indicate that compositional factors explain the healthy migrant effect (7). Studies focusing on other health measures instead of mortality on middle-aged and older people find worse health outcomes among the foreign-born than the native populations [e.g., (8–10)].

Cognitive functioning has been proposed as one of the indicators of population aging due to the increasing role of cognition in societies that have experienced transformations of work and social life (11). In aging populations, it is important to understand the scope and healthcare needs of a potentially increasing share of people with cognitive functioning issues or dementia, as well as which population groups are most affected. Previous research findings are still inconsistent regarding the foreign-origin population’s position in cognitive functioning with respect to other population groups (12, 13). Studies that compared the cognitive functioning of migrants to their origin country population, or both the origin as well as the host country populations, found either that migrants have worse cognition [e.g., (14, 15)], or that the migrants do not differ from non-migrants in terms of cognitive functioning.

Age, language skills, and education are strongly associated with cognitive functioning (12, 15–17). Educational levels and quality are usually reflected in socioeconomic status (SES); low SES is likely to be associated with worse cognitive performance, but also with decreased access to healthcare services, which affects cognitive functioning negatively (12, 17). This negative link with SES has been mostly confirmed for Hispanic people in the North American context, but not in other contexts (12). Furthermore, chronic health conditions, lifestyle factors, and adverse experiences vary between the different origin groups, which also influence cognitive functioning (*ibid*). Age at migration has been found to matter as well – those who migrated to the US in mid-life (ages 20–49) tend to show better cognitive functioning, especially men, who indicate a slower cognitive decline than women (13), possibly due to gender differences in education, stress or access to healthcare. Finally, early life conditions (e.g., parental education) may mediate some life pathways, thereby also influencing later life cognitive performance (12). There are several measurement issues to be considered, which become pertinent when comparing different origin groups (14). Some authors use specially adjusted cognitive functioning measures when comparing different populations, because normative measures that have developed in Western countries may erroneously result in false-positive outcomes (14, 16).

As Europe has become one of the main immigration destinations over the last few decades, migrants and their descendants now form 18 percent of the European Union population. Among them, 52 percent are from outside Europe. With a migrant community of 1.8 million, Russians constitute a sizable foreign-origin group in Europe (18). Estonia ranks third highest in Europe for the proportion of its foreign-born population and their descendants (33 percent), and first for the share of the second generation foreign-origin population group (21.5 percent) (18, 19). The majority of these migrants and their descendants are Russians, as this population has been in formation since the post-World War II decades when Estonia was incorporated into the Soviet Union (20). This strengthens our motivation to focus on the Russian-origin population in Estonia, comparing them to Estonians in Estonia and Russians in Russia to provide a unique design setting not yet tested elsewhere. The general societal features in the lives of the current middle-aged and older people in Estonia and Russia, as well as the migration, were quite different from those in Western countries, making it an interesting case.

The migration policies of the Soviet Union created incentives to move for labor reasons, which was facilitated by centralized policies. Migrants received housing, and were favored due to belonging to the labor force needed for economic development (especially construction, industry, and government employees), and because housing was a deficit product (21).

Due to the large migration turnover, only about 11 percent of migrants remained in Estonia by the beginning of the 1990s (22). Only about 4 percent of the current foreign-origin population arrived in Estonia after regaining independence in 1991 (23). The age structure of the foreign-origin population was relatively young compared with the native Estonian population due to the constant inflow of migrants up until the 1990s (24). The educational structure of people who remained in Estonia displays equivalent levels to those of the native Estonian population (23). However, despite their similar educational levels, the foreign-origin population was often employed in fields that were better paid, but required fewer qualifications (25), due to the Soviet specificity of working-class preferences (e.g., wage differences were in the favor of industrial and agricultural workers) (26). Additionally, the health of the migrant population in Estonia has been consistently worse than that of the native population, mostly due to health behavior differences (27, 28).

Both Estonia and Russia experienced life expectancy stagnation for over 40 years since the 1960s, although this tendency was particularly noticeable among men in Russia. In the 1970s, the trend for non-Estonian men followed that of Russian men. After 1998, however, the trend followed that of Estonian men (23). By 2009, the life expectancy of non-Estonian men in Estonia averaged at 67 years, and 79.4 for non-Estonian women, remaining between Estonians in Estonia and Russians in Russia (19, 29). The large gender gap in life expectancy in both countries is caused by the excessive mortality of working-age men, with the most widespread causes of death being cardiovascular diseases, followed by external causes such as accidents and violence (23, 27, 30). Consequently, the health-adjusted life expectancy (HALE) has been low in both Estonia and Russia relative to other European countries, although the Estonian HALE is higher than in Russia. In 2010, the Russian male HALE was 56.2 years from birth, and 11.0 years from the age of 60, which was, respectively, 7.1 and 2.4 years less than for men in Estonia. The Russian female HALE was 64.9 years from birth, and 15.1 years from the age of 60, which are 5.3 and 2.8 years less than for women in Estonia (30).

The healthcare systems of both countries had common features during the Soviet period of 1945–1991, with a focus on infectious diseases, poor technological equipment, and insufficient training to address emerging chronic and age-related illnesses. Even though the general governing principles were similar from the post-World War II decades until 1991, economic recovery since then as well as improvements in the social and healthcare spheres were faster in Estonia than in Russia. A distinct feature in the case of Estonia is that much of the education, media, social and healthcare services remained available in Russian after 1991, making it possible for Russians in Estonia to continue using them in their own language. Healthcare expenditure constituted 7 percent of the Estonian GDP, and 5.4 percent in Russia in 2009 (31). The share of out-of-pocket (OOP) payments increased in the 1990s in both countries, but reached 20 percent in Estonia by the 2000s, and almost 29 percent in Russia by 2009 (32, 33). Most of these costs were spent on pharmaceuticals, affecting vulnerable population groups the most (34). The reduced availability of free healthcare and drug therapy among the older population in Russia may be responsible for unmet medical assistance leading to the onset of chronic diseases, including the development of cognitive functioning (35).

Ten years ago, both countries lacked an effective healthcare system for age-related diseases and long-term care. However, Estonia became better prepared to address the challenges of dementia compared with Russia by launching a systemic healthcare reform at the end of the 1990s, developing nursing care homes, and increasing the availability of psychiatrists and support from family doctors (36, 37). The massive development of outpatient geriatric care aimed at monitoring and caring for patients, as well as providing supportive therapy, began in Russia only in 2017. Until then, older adults with self-care problems (including from mental disorders) only received care services on the shadow market or from relatives. Mental healthcare was provided with poor-quality services for people with severe mental disorders, and was not focused on prevention or treatment (33).

In Russia, the proportion of 60+ people with dementia (4.9 percent) was lower than in Estonia (5.3 percent), remaining below the OECD average in both countries in 2009 (38). This can be explained by relatively low life expectancy (not many people survive up to their dementia) and insufficient disease detection due to individual and public attitudes regarding dementia as a mental disorder (39). However, the dementia prevalence is expected to increase to 17 percent in Russia, and 26 percent in Estonia by 2050, given that the current health trends continue (32).

This paper’s main aim is to identify the proportion of middle-aged and older people that are at risk of (mild) cognitive functioning problems in Estonia and Russia, and to understand which factors are associated with cognitive functioning of the non-institutionalized middle-aged and older population, comparing the Russian-origin population in Estonia with Estonians in Estonia and Russians in Russia. We also distinguish Russians in Estonia based on their age at migration. Such a design enables us to consider the possible effects of different selection criteria, age structure differences, and the role of (dis)advantages in later-life health when studying migration or migrant effects on population health. Since the migration event itself took place a relatively long time ago in the lives of these individuals, we can estimate long-term effects (and are less concerned with the immediate effects of the move itself). Given the generally worse health and mortality indicators among Russians in Estonia as well as in Russia, and the lower dementia prevalence in Russia, we expect that the cognitive functioning outcomes of Russians in Estonia remain in between those of Estonians and Russians.

## 2. Materials and methods

### 2.1. Samples

We use data from two surveys aimed at studying individual aging – SHARE (Survey of Health, Ageing and Retirement in Europe), and SAGE (The Study on Global Ageing and Adult Health). Both targeted people aged 50+, and included the partners of the main respondents. The first wave of the Estonian SHARE survey was carried out from 2010 to 2011. The sample frame of SHARE Estonia was based on the national population register, which selected age-eligible target individuals from each household. Stratified sampling was used with a simple random sampling of individuals within the strata. Stratification was done by gender and year of birth. Within each gender-age stratum, records are sorted by region for better geographical allocation. Prior to the fieldwork, the sample was double-checked with the death register to exclude any possible deaths that occurred after the sampling. The household response rate for Estonia was close to 60 percent (40). The language of the SHARE Estonia survey depended on the respondents’ preference – it was conducted in Estonian or in Russian, with most of the Russians in Estonia choosing to respond in Russian. Therefore, the words used in the cognitive functioning measures were also different, depending on the language of the survey. The words used in the cognitive functioning measurement in the Russian language questionnaire were somewhat different from the Russian SAGE survey’s cognitive functioning items. However, they reflect different spheres of daily speech (10), and were developed by local psychologists based on the international measures (41).

The first wave of the SAGE Russian survey was carried out from 2007 to 2010. The national sample was constructed using data from two sources: the sample for the 2003 World Health Survey (WHS) and the 2002 population census. The aim of the sampling design was to obtain a nationally representative cohort of people aged 50 years and older, with a smaller cohort of people aged 18 to 49 for comparison. In this study, we use data on people aged 50 and above. The total individual response rate for SAGE was 71.8 percent (42).

For the purpose of this paper, we chose people living in urban areas, as over 90 percent of Russians in Estonia have settled there, so choosing only urban dwellers helps to reduce possible selection effects on area of residence.^1^ In addition, we ran analyses distinguishing Russians in Estonia by whether they were born in Estonia or not, and by age at migration, but the number of cases in some of these groups was too small to make reliable conclusions.^2^^,^^3^ Results from these additional analyses based on differently defined groups are presented in Supplementary material.

We distinguish migrant groups by self-reported ethnicity, as this is comparable in both surveys, by including Estonians in Estonia, Russians in Estonia (first- and second-generation) and Russians in Russia. Furthermore, we included only Estonians who were born in Estonia and whose mother was born in Estonia. The Russian SAGE survey made it possible to distinguish those who had been living most of their adulthood or childhood abroad – so we did not include them in our analytical sample in order to reduce other potential migration effects. This leaves us with 2,365 Estonians, 1,373 Russians in Estonia, and 2,339 Russians in Russia (total *N* = 6,077). An additional analysis of groups defined based on age at migration distinguished Russians in Estonia into following groups: those who were born in Estonia (*n* = 238), those who moved to the country as children before age 18 (*n* = 330), those who moved there between ages 18–24 (*n* = 438), and those who moved there at age 25 or later (*n* = 367). The age profiles of these groups are presented in Supplementary Figure S1.

### 2.2. Variables

We looked at the following cognitive functioning outcomes – verbal fluency and verbal recall, which measure and reflect the memory domain, and semantic fluency in the domain of cognition. It may be difficult to distinguish when cognitive impairment is a manifestation of dementia or a serious clinical condition from when it is a normal age-related effect (43). Some studies suggest using group-associated percentile-based cut-off thresholds to indicate cognitive impairment in order to reflect people with a serious clinical condition (43, 44).

Verbal fluency refers to the ability to produce as many words as possible in a one-minute time span, assessing information retrieval from semantic memory and measuring crystallized knowledge accumulated over an extended period of time. According to SHARE criteria, a score of less than 18 items represents impairment in word fluency (45). Immediate and delayed recall assess learning capacity, memory storage and memory retrieval, measuring temporary working memory that is more prone to be affected by aging compared to crystallized memory (46). These are tested by presenting 10 words, after which the respondent is given the opportunity to recall as many words as possible. In SAGE, this was repeated three times to saturate the learning curve, while in SHARE, this was repeated two times. After about 5 min in SHARE and 10 min in SAGE, delayed recall and recognition were tested again. According to SHARE criteria, a score of 4 or fewer words represent impairment in verbal learning and recall (45).

The measurement of verbal recall (or immediate recall) is most similar in both Estonian and Russian surveys. The measurement of delayed recall was slightly different; in the SHARE survey, a list of words was asked to be repeated two times, while in the SAGE survey, it was asked to be repeated three times, but each time before repetition the list was given anew (see Supplementary material for the exact question formulations and word list differences). While the fluency test was seemingly similar in both surveys, the outcomes are two times lower for the indicator in SAGE than in SHARE, indicating unidentified measurement differences. In order to achieve comparable measures for both surveys, we used a 25-percentile-based cut-off threshold for each group and for both cognitive functioning outcomes separately, following some examples from other studies (43, 44). As a result, the cut-off points for fluency appear below the suggested international thresholds. However, we believe that this allows for a more suitable comparison. Due to comparability issues, we present results only for two outcomes - fluency and immediate recall.

Binary logistic regression models are run separately for men and women. Control variables that were asked in both surveys were included step by step, and cover the most relevant factors, such as demographic, socioeconomic, psychosocial, health, and health behavior, chosen based on existing literature findings (12, 47). Specifically, these include age (at time of interview), marital status (married or partnered/separated or divorced/widowed/never married), total years spent in education, employment status (in employment/retired/other such as at home or ill), evaluation of respondents’ current financial situation (having difficulties/not), ownership status of dwelling (owner/other), self-rated health ((very) bad/fair/(very) good), depressiveness, smoking and alcohol consumption, BMI (NA/ <18.5/18.6–22.9/ 23–24.9/25–29/ 30–34.9/35+), satisfaction with personal relations, trust in people, receipt of care/help, mother’s education (NA/below secondary/secondary or highest) and father’s education (NA/below secondary/secondary or highest). The comparison of the questions and response options that have differed in the surveys of the two countries and their transformation for the current analysis are presented in Table 1 and Supplementary Table S5.

**Table 1:** Descriptive results for the cognitive functioning outcomes of different population groups aged 50+, SHARE Estonia 2010–2011, and SAGE Russia 2007–2010.

	Estonians		Russians in Estonia		Russians in Russia	
	Women (*N* = 1,431)	Men (*N* = 934)	Women (*N* = 843)	Men (*N* = 530)	Women (*N* = 1,579)	Men (*N* = 760)
Verbal fluency (Mean (CI))	22.1 (21.7–22.5)	21.5 (21.0–21.9)	20.2 (19.7–20.7)	19.7 (19.1–20.4)	12.1 (11.7–12.4)	12.2 (11.6–12.7)
Immediate recall (Mean (CI))	5.5 (5.4–5.4)	5.0 (4.9–5.1)	5.0 (4.9–5.1)	4.6 (4.4–4.8)	5.2 (5.1–5.3)	5.3 (5.2–5.4)
						
Fluency impairment (%)	20.61	21.63	20.76	21.70	23.81	23.82
Immediate recall impairment (%)	11.18	16.81	20.40	24.34	13.55	14.08

## 3. Results

### 3.1. Descriptive results

Descriptive statistics of the three population groups are presented in Tables 1, 2. Russians in Estonia are on average slightly younger than Estonians, but older than Russians in Russia (men 66.5 years, women 67.5 years on average). If different migrant generations were separated, the first generation would be the oldest group, while the second generation would be the youngest group by almost 10 years.

**Table 2:** Descriptive results for different population groups aged 50+, SHARE Estonia 2010–2011, and SAGE Russia 2007–2010.

		Estonians				Russians in Estonia				Russians in Russia		
		Women (*N* = 1,431)		Men (*N* = 934)		Women (*N* = 843)		Men (*N* = 530)		Women (*N* = 1,579)		Men (*N* = 760)
	*N*	Mean (CI)/%	*N*	Mean (CI)/%	N	Mean (CI)/%	*N*	Mean (CI)/%	*N*	Mean (CI)/%	*N*	Mean (CI)/%
Age (mean)		68.5 (68.0–69.0)		67.5 (66,9–68.1)		67.5 (66.8–68.2)		66.5 (65.7–67.4)		66.6 (66.1–67.1)		64.5 (63.8–65.2)
Years of education (mean)		12.0 (11.8–12.2)		11.9 (11.7–12.1)		11.2 (10.9–11.4)		11.5 (11.2–11.8)		11.2 (11.0–11.3)		11.5 (11.2–11.7)
												
Married/partnered	691	48.29	728	77.94	455	53.97	445	83.96	650	41.17	579	76.18
Separated/divorced	229	16.00	91	9.74	124	14.71	50	9.43	159	10.07	62	8.16
Widowed	383	26.76	58	6.21	232	27.52	22	4.15	718	45.47	106	13.95
Never married	128	8.94	57	6.10	32	3.80	13	2.45	51	3.23	13	1.71
												
In employment	528	36.90	395	42.29	254	30.13	203	38.30	463	29.32	303	39.87
Retired	845	59.05	488	52.25	541	64.18	300	56.60	954	60.42	354	46.58
Other	58	4.05	51	5.46	48	5.69	27	5.09	149	9.44	101	13.29
												
Living alone (%)	507	35.43	113	12.10	267	31.67	56	10.57	581	36.80	132	17.37
												
Difficulties with economic situation	114	7.97	49	5.25	203	24.08	63	11.89	499	31.60	180	23.68
												
Own dwelling	940	65.69	460	49.25	576	68.33	242	45.66	1,438	91.07	692	91.05
												
Self-rated health: (very) bad	340	23.76	264	28.27	309	36.65	142	26.79	512	32.43	181	23.82
Self-rated health: fair	678	47.38	412	44.11	415	49.23	266	50.19	914	57.88	435	57.24
Self-rated health: (very) good	412	28.79	256	27.41	119	14.12	121	22.83	151	9.56	141	18.55
												
Depressed	574	40.11	270	28.91	456	54.09	170	32.08	769	48.70	240	31.58
												
BMI: DK/NA/R	34	2.38	15	1.61	25	2.97	5	0.94	50	3.17	25	3.29
BMI: <18.5	22	1.54	5	0.54	7	0.83	5	0.94	22	1.39	11	1.45
BMI: 18.5–22.9	216	15.09	118	12.63	101	11.98	85	16.04	145	9.18	99	13.03
BMI: 23.0–24.9	233	16.28	187	20.02	93	11.03	101	19.06	180	11.40	132	17.37
BMI: 25.0–29.9	530	37.04	399	42.72	320	37.96	221	41.70	601	38.06	359	47.24
BMI: 30.0–34.9	292	20.41	154	16.49	185	21.95	84	15.85	367	23.24	104	13.68
BMI: 35.0 +	104	7.27	56	6.00	112	13.29	29	5.47	214	13.55	30	3.95
												
Currently smoking	152	10.62	248	26.55	94	11.15	172	32.45	76	4.81	320	42.11
												
Alcohol drinking: DK/NA/R	2	0.14	0	0.00	1	0.12	4	0.75	439	27.80	79	10.39
Alcohol: Never	678	47.38	261	27.94	424	50.30	152	28.68	291	18.43	91	11.97
Alcohol: Sometimes	646	45.14	310	33.19	384	45.55	233	43.96	825	52.25	457	60.13
Alcohol: Often	105	7.34	363	38.87	34	4.03	141	26.60	24	1.52	133	17.50
												
Satisfaction with relations: DK/NA/R	29	2.03	36	3.85	16	1.90	28	5.28	21	1.33	14	1.84
Satisfaction with relations: dissatisfied	11	0.77	26	2.78	22	2.61	5	0.94	98	6.21	31	4.08
Satisfaction with relations: neutral/fair	75	5.24	57	6.10	40	4.74	38	7.17	242	15.33	81	10.66
Satisfaction with relations: satisfied	1,315	91.89	815	87.26	765	90.75	459	86.60	1,203	76.19	630	82.89
												
Trust in people: DK/NA/R	26	1.82	38	4.07	15	1.78	28	5.28	16	1.01	7	0.92
Trust in people: no/low trust	183	12.79	128	13.70	88	10.44	89	16.79	465	29.45	254	33.42
Trust in people: (high) trust	1,222	85.39	768	82.23	740	87.78	413	77.92	1,085	68.71	495	65.13
												
Receipt of care: DK/NA/R	161	11.25	195	20.88	68	8.07	109	20.57	19	1.20	9	1.18
Receipt of care: no	933	65.20	584	62.53	589	69.87	345	65.09	1,196	75.74	630	82.89
Receipt of care: yes	337	23.55	155	16.60	186	22.06	76	14.34	362	22.93	119	15.66
												
Education of mother: DK/NA/Other	295	20.61	259	27.73	351	41.64	225	42.45	77	4.88	49	6.45
Education of mother: below secondary	828	57.86	473	50.64	331	39.26	207	39.06	1,078	68.27	493	64.87
Education of mother: secondary or more	308	21.52	202	21.63	161	19.10	98	18.49	424	26.85	218	28.68
												
Education of father: DK/NA/Other	374	26.14	291	31.16	339	40.21	227	42.83	200	12.67	75	9.87
Education of father: below secondary	730	51.01	429	45.93	305	36.18	184	34.72	894	56.62	414	54.47
Education of father: secondary or more	327	22.85	214	22.91	199	23.61	119	22.45	485	30.72	271	35.66

Immediate recall and fluency averages are generally slightly higher among women than men, except for Russians in Russia (Table 1). Among men, the average immediate recall is lowest among Russians in Estonia (4.6 words), and highest among Russians in Russia (5.3 words). Among women, Russians in Estonia also have the lowest average immediate recall (5.0 words), followed by Russians in Russia, and lastly Estonians (5.5 words). The gender differences in immediate recall are significant for Estonians and Russians in Estonia, but not for Russians in Russia. Mean fluency scores are also lower for Russians in Estonia than Estonians among both women and men. The gender differences in fluency scores are not significant for any group.

Due to some measurement differences, looking at the proportion of impaired people gives a better overview from a comparative perspective. Among both women and men, Russians in Russia have the largest proportion of people with impaired fluency (Table 1). Russians in Estonia have the highest share of people impaired in immediate recall, while Russians in Russia and Estonian women have the lowest.

### 3.2. Immediate recall

Results for immediate recall models are presented in Table 3. The unadjusted binary logistic regression model for verbal learning (immediate recall) (Model 0 in Table 4) indicated that the odds of impairment for Russian men in Estonia were almost double those of Russian men in Russia (OR 0.51, 95% 0.38–0.69), and the odds of impairment were about 1.5 times compared with Estonian men in Estonia (OR 0.66, 95% 0.51–0.87). For women, the unadjusted odds were doubled among Russians in Estonia than Estonian women (OR 0.51, 95% 0.40–0.64), and 1.6 times higher than for Russians in Russia (OR 0.62, 95% 0.50–0.78). The included variables reduced the group differences, but not compared with Estonians. Finally, adjusted regression models for immediate recall (Model 8 in Table 4) showed that Russians in Estonia have significantly higher odds of cognitive impairment compared with Estonians among both men (OR 0.68, 95% 0.49–0.92) and women (OR 0.62, 95% 0.46–0.82). Final impairment odds for men remained about 1.5 times higher for Russian men in Estonia compared with Estonian men, and 1.3 times higher compared with Russian men in Russia (OR 0.78, 95% 0.52–1.17), the latter being not significantly different. Final impairment odds for women remained 1.6 times higher for Russians in Estonia compared with Estonian women, and 1.2 times higher compared with Russian women in Russia (OR 0.81, 95% 0.58–1.13), the latter being once again not significant.

**Table 3:** Coefficients of impairment in immediate recall from binary logistic regression models for different population groups aged 50+, SHARE Estonia 2010–2011, and SAGE Russia 2007–2010.

		M0	M1	M2	M3	M4	M5	M6	M7	M8
		OR (95% CI)	OR (95% CI)	OR (95% CI)	OR (95% CI)	OR (95% CI)	OR (95% CI)	OR (95% CI)	OR (95% CI)	OR (95% CI)
Men (ref: Russians in Estonia)	Estonians	0.663 (0.505–0.872)**	0.598 (0.449–0.797)***	0.576 (0.431–0.769)***	0.624 (0.465–0.838)**	0.630 (0.469–0.847)**	0.634 (0.470–0.855)**	0.625 (0.462–0.848)**	0.630 (0.464–0.856)**	0.675 (0.494–0.922)*
	Russians in Russia	0.514 (0.382–0.691)***	0.568 (0.416–0.776)***	0.562 (0.408–0.773)***	0.496 (0.358–0.689)***	0.580 (0.407–0.828)**	0.596 (0.417–0.852)**	0.619 (0.426–0.900)*	0.651 (0.444–0.956)*	0.780 (0.519–1.172)
									
	*N*	2096	2096	2096	2096	2096	2096	2096	2096	2096
	AIC	1918.545	1748.468	1749.166	1701.338	1699.497	1681.607	1693.092	1692.972	1689.383
	BIC	1935.488	1771.059	1794.348	1763.463	1772.918	1771.972	1839.935	1879.349	1898.351
	*R*^2^	0.0101	0.0992	0.1030	0.1308	0.1338	0.1462	0.1506	0.1579	0.1639
		M0	M1	M2	M3	M4	M5	M6	M7	M8
		OR (95% CI)	OR (95% CI)	OR (95% CI)	OR (95% CI)	OR (95% CI)	OR (95% CI)	OR (95% CI)	OR (95% CI)	OR (95% CI)
Women (ref: Russians in Estonia)	Estonians	0.506 (0.397–0.643)***	0.423 (0.327–0.546)***	0.419 (0.323–0.541)***	0.494 (0.380–0.643)***	0.516 (0.395–0.674)***	0.579 (0.441–0.760)***	0.566 (0.430–0.744)***	0.559 (0.425–0.737)***	0.616 (0.462–0.821)**
	Russians in Russia	0.624 (0.497–0.783)***	0.630 (0.495–0.803)***	0.602 (0.469–0.772)***	0.559 (0.434–0.722)***	0.579 (0.446–0.752)***	0.609 (0.467–0.794)***	0.708 (0.529–0.946)*	0.664 (0.491–0.899)**	0.809 (0.582–1.125)
									
	*N*	3,712	3,712	3,712	3,712	3,712	3,712	3,712	3,712	3,712
	AIC	3891.041	3558.794	3552.816	3435.214	3436.105	3425.209	3375.805	3376.062	3361.410
	BIC	3909.699	3583.671	3602.570	3503.627	3516.957	3524.718	3537.508	3581.300	3591.525
	*R*^2^	0.0105	0.1283	0.1298	0.1618	0.1661	0.1796	0.1847	0.1904	0.1970

Variables entered step by step in the following order: M0, observation group; M1, age; M2, household size, marital status; M3, employment status, years spent in education; M4, self-reported economic situation, ownership status of dwelling; M5, self-rated health, depressiveness; M6, tobacco consumption, alcohol consumption; BMI: M7, satisfaction with relationships, trust in people, receipt of (care) assistance; M8, mother’s education, father’s education. ***p < 0,001; **p < 0.01; *p < 0.05.

**Table 4:** Coefficients of impairment in verbal fluency from binary logistic regression models for different population groups aged 50+, SHARE Estonia 2010–2011, and SAGE Russia 2007–2010.

		M0	M1	M2	M3	M4	M5	M6	M7	M8
		OR (95% CI)	OR (95% CI)	OR (95% CI)	OR (95% CI)	OR (95% CI)	OR (95% CI)	OR (95% CI)	OR (95% CI)	OR (95% CI)
										
Men (ref: Russians in Estonia)	Estonians	1.000 (0.766–1.304)	0.959 (0.731–1.258)	0.894 (0.679–1.777)	0.984 (0.744–1.301)	0.995 (0.752–1.317)	1.000 (0.753–1.329)	0.968 (0.724–1.293)	0.954 (0.711–1.280)	0.988 (0.733–1.332)
	Russians in Russia	1.086 (0.827–1.428)	1.217 (0.920–1.611)	1.172 (0.879–1.561)	1.102 (0.822–1.476)	1.086 (0.794–1.487)	1.121 (0.817–1.538)	1.041 (0.744–1.457)	0.951 (0.675–1.340)	1.036 (0.720–1.489)
									
	*N*	2096	2096	2096	2096	2096	2096	2096	2096	2096
	AIC	2243.903	2158.761	2132.775	2077.364	2079.876	2060.517	2024.291	2008.262	1999.366
	BIC	2260.846	2181.352	2177.957	2139.489	2153.297	2150.882	2171.133	2194.639	2208.334
	*R*^2^	0.0003	0.0392	0.0544	0.0818	0.0825	0.0938	0.1189	0.1323	0.1399
		M0	M1	M2	M3	M4	M5	M6	M7	M8
		OR (95% CI)	OR (95% CI)	OR (95% CI)	OR (95% CI)	OR (95% CI)	OR (95% CI)	OR (95% CI)	OR (95% CI)	OR (95% CI)
Women (ref: Russians in Estonia)	Estonians	0.980 (0.792–1.213)	0.907 (0.725–1.134)	0.897 (0.717–1.123)	1.063 (0.844–1.339)	1.094 (0.867–1.381)	1.177 (0.929–1.490)	1.174 (0.923–1.494)	1.178 (0.924–1.502)	1.228 (0.954–1.580)
	Russians in Russia	1.176 (0.957–1.446)	1.272 (1.025–1.581)*	1.205 (0.966–1.503)	1.166 (0.930–1.461)	1.159 (0.921–1.459)	1.185 (0.939–1.495)	1.119 (0.863–1.450)	1.090 (0.834–1.425)	1.223 (0.917–1.632)
									
	*N*	3,712	3,712	3,712	3,712	3,712	3,712	3,712	3,712	3,712
	AIC	3891.041	3558.794	3552.816	3435.214	3436.105	3425.209	3375.805	3376.062	3361.410
	BIC	3909.699	3583.671	3602.570	3503.627	3516.957	3524.718	3537.508	3581.300	3591.525
	*R*^2^	0.0012	0.0871	0.0907	0.1225	0.1233	0.1277	0.1455	0.1490	0.1549

Variables entered step by step in the following order: M0, observation group; M1, age; M2, household size, marital status; M3, employment status, years spent in education; M4, self-reported economic situation, ownership status of dwelling; M5, self-rated health, depressiveness; M6, tobacco consumption, alcohol consumption, BMI; M7, satisfaction with relationships, trust in people, receipt of (care) assistance; M8, mother’s education, father’s education. ***p < 0.001; **p < 0.01; *p < 0.05.

Although the odds of impairment for Russians in Estonia were initially also significantly higher than those of Russians in Russia, the differences decreased among both men and women after controlling for health behavior and social factors (Models 6 and 7), and disappeared completely after adjusting for parental education (Model 8). In general, none of the included variables explained much of the impairment differences, remaining between 16.4 percent and 19.7 percent depending on the population group. The variables did explain more differences for women than men, however, as well as compared with Russians in Russia than with Estonians.

Therefore, Russians in Estonia have worse cognitive functioning than other observation groups regarding the immediate recall measure, but this factor remains significantly worse when compared with Estonians after all variables have been adjusted for.

Interaction models with gender (not presented here) indicate that men have 1.4–1.7 times higher odds of impairment than women among all groups.

Additional analyses distinguishing groups based on their age at migration indicate no differences between the groups in immediate recall outcomes (Supplementary Table S7).

### 3.3. Fluency

For the fluency indicator, Russians in Estonia overall had lower odds of impairment than Estonians and Russians in Russia among women, and lower odds of impairment than Russians in Russia among men, but none of these differences were significant (Table 4). Additionally, the odds of impairment compared with Estonian men did not differ. The significantly higher impairment in fluency among Russian women in Russia compared with Russian women in Estonia emerged after controlling for age (Model 1 in Table 4), but disappeared again after controlling for other demographic factors such as marital status and household size (Model 2). In all other cases, none of the variables included in the models changed the position or the significance level of the odds of impairment.

The included variables explained little of the impairment differences between groups, even less than in the case of immediate recall – at 14 percent for men and 15.5 percent for women – and the variables explained more differences among women than men.

Interaction models with gender (not presented here) indicated that the odds of impairment for men were 1.3–1.6 times higher than women among all groups. Additional analyses distinguishing groups based on their age at migration indicated no differences between the groups in immediate recall outcomes (Supplementary Table S7).

Descriptive and regression results of verbal recall and fluency impairment for men and women according to different analytical groups that distinguish Russians in Estonia by whether they were born in Estonia, or at which age they migrated to Estonia (as specified in footnote 3) are presented in the Supplementary material.

## 4. Discussion

As cognitive functioning is an increasingly relevant aspect of health in aging societies, it is important to identify its main predictors and groups at risk. Working memory is a temporary type of memory, and shows deficiencies when a person ages more clearly than crystallized knowledge (46). Therefore, it can be used as a predictor for dementia onset. Based on both verbal learning and fluency measures observed in this study, we conclude that the minimum share of older people at risk of impairment is around 20–24 percent among the middle-aged and older foreign-origin population in Estonia, and based on the immediate recall indicator, it is about 11–17 percent for Estonians and 13–14 percent for Russians in Russia. In the case of fluency, it reaches above 20 percent for both. These shares are close to the total population predicted estimates for Estonia and Russia by 2050 (32), and so they may slightly overestimate the actual share of middle-aged and older people with a clinical condition. Our findings are probably a better approximation of the share of middle-aged and older people with at least some (including milder) cognition problems rather than severe dementia, and would need further predictive modelling in the future to assess the reliability of the projections for these countries provided elsewhere.

Contrary to the expectations of the healthy migrant effect, we find that foreign-origin population groups in Estonia have the highest risk of cognitive health impairment. However, this only holds for the immediate recall outcome, and when age at migration is not considered. The slightly advantaged position in fluency can be considered comparable with the fluency outcome of non-migrants, due to not reaching statistically significant levels in any of the models. The first conclusion is in line with previous findings regarding Estonian and Russian epidemiological and mortality developments, showing that the health and life expectancy of the foreign-origin population is worse than that of Estonian native population (23, 27, 30, 48). However, it also confirms previous results on migrant health in other international settings that have studied middle-aged and older populations, including a multi-country design setting, or analyses of different health outcomes besides mortality [e.g., (6, 8–10)], such as cognition (14, 15). The outcome for fluency shows somewhat novel findings for this geographical and social setting, and confirms findings where no differences in cognitive functioning between migrants and non-migrants have been found [e.g., (16)]. These outcomes call for a more thorough analysis on these population groups using a broader set of cognitive functioning indicators, when these become available.

Somewhat contradictory findings for Russians in Estonia between fluency and immediate recall indicate that the effect of the migration experience may differ depending on the cognitive functioning outcomes. Crystallized knowledge, which fluency measures, does not seem to be affected by the migration experience, unlike working memory. One possible explanation is that since the middle- and older-generation Russians in Estonia did not find it necessary to learn Estonian, due to the accessibility of Russian for daily activities and services, their language-learning skills were left inactive, which may have had a detrimental effect on verbal recall (13). People with multiple language skills may have better cognitive functioning, even if not formally educated (41, 49).

Our additional analyses suggest that the observation groups do not differ in verbal recall outcomes when age at migration is considered in defining the migrant groups, and those born in Estonia were excluded from the analysis due to the small sample size of this group. This would further strengthen support for the other reported findings (16), and also support the relevance of accounting for age at migration and age profiles of different foreign-origin groups for cognitive health (13). Migrants have moved to Estonia across different life stages, in childhood as much as during the most active migration years (ages 18–24) and in adulthood. While those who moved after the age of 25 show a somewhat larger proportion of impairment in both cognitive functioning outcomes, they are also on average older than other respondents, which explains why regression results do not indicate any differences in cognitive functioning impairment between the observation groups.

The fact that cognitive functioning among Russians in Russia is not generally worse than that of Russians in Estonia contradicts earlier findings on health in the region. This recent outcome suggests that due to long-term higher mortality rates in Russia compared with Estonia, the older population has become selective in terms of (cognitive) health, and that only those with better health have survived to this age and participated in the survey. When life expectancy rises in Russia to levels comparable to other countries, this cognitive advantage will likely disappear.

Unlike many previous studies, there was a higher average of education levels among women compared with men for most of the population groups, except for Russians in Russia, indicating some selection effects. Better-educated men have survived longer, and are therefore more likely to be survey respondents. Also, among all population groups, the proportion of impaired people is lower among women than men for both immediate recall and fluency. This is reflected in a higher risk of impairment among men for most observed groups, which is again a somewhat different finding compared with usual reports of men performing better than women in cognitive functioning [e.g., (13, 16)]. This potentially intriguing gender finding should be further studied in the future, as this aspect was not the purpose of this article. Our findings confirm the important role of an individual’s own education and parental socioeconomic status in later life cognitive functioning (12, 17). Some health behavior differences between Russians in Russia and Russians in Estonia accounted for recall impairment differences, also confirming some of the earlier explanations on the development of illnesses and causes of death in both countries (23, 27, 30).

This article can be used to argue for the need to increase investment and attention on the prevention and diagnosis of cognitive functioning in these regions, including addressing the people’s general attitude toward dementia and other mental illnesses (39), to better identify risk factors for the different groups.

## 5. Strengths and limitations

This study uses the similar measures from two surveys on aging as a unique opportunity to compare foreign-origin population groups in both the host country and the origin country population. This allowed for the consideration of potential selection and age structure effects, as well as differences in economic and social circumstances. We contribute to the literature on migration and health by considering the long-term effects of migration on later life cognitive functioning, finding that the differences vary or are non-existent depending on the cognition outcome and how the compared groups are structurally defined. We also differentiated the foreign-origin population group by their place of birth (i.e., distinguishing the first- and second-generation), and by their age at migration, which may have taken place during different life periods, potentially influencing cognitive functioning. This is still an understudied aspect in (cognitive) health outcomes. Our findings indicate that these structural factors are relevant in explaining group differences, but more studies in future with larger sample sizes by group and by gender are needed in order to be able to make more conclusive interpretations.

Some differences in the measurement potentially influence results. First, the language of the survey was conducted in the preferred language of the respondent in Estonia – either in Estonian or Russian, and language differences are a well-known influencing factor in cognitive functioning. Second, although Russians in both Estonia and in Russia replied to the questionnaire in Russian, using cognitive functioning measures in the Russian language, the recall lists included different words (see Supplementary material), potentially influencing their capability of memorization. We have not been able to adjust for the language of the interview (which was not recorded properly in SHARE Estonia Wave 4 due to technical problems) or for language skills such as bilingualism. The latter tends to have a positive effect on cognitive functioning, even among people who are not formally educated (41, 49), so future studies on this topic and this regional context could also focus more on the language factors. Finally, although we included only variables from both surveys that were possible to transform into comparable data, some measurement differences may affect the outcomes.

Selection issues must be considered as well. The higher mortality of Russian men causes a large gender gap in life expectancy, which might have resulted in a more selective survival of Russians in Russia compared with Russians or Estonians in Estonia. Therefore, the sample of SAGE is also potentially selective, with people in better health being more likely to be included as respondents of the survey.

## Data availability statement

Publicly available datasets for researchers were analyzed in this study. This data can be found at: https://share-eric.eu/data/data-access. All data used in our study are available free of charge to all scientific users world-wide after individual registration (https://share-eric.eu/data/become-a-user). SHARE data are DOI registered datasets. Each wave and each release is assigned a persistent DOI. In our article, we use SHARE data from Wave 4 (DOI: 10.6103/SHARE.w4.800). Russian WHO SAGE Wave 1 data is available at: https://apps.who.int/healthinfo/systems/surveydata/index.php/catalog/68 (DDI-RUS-WHO-SAGE-2007-V01).

## Ethics statement

The studies involving human participants were reviewed and approved by the Ethics Committee of the University of Mannheim and Ethics Council of the Max Planck Society. For more details see: https://share-eric.eu/fileadmin/user_upload/Ethics_Documentation/SHARE_ethics_approvals.pdf. The participants provided their written informed consent to participate in this study.

## Author contributions

LA analyzed the data and wrote the manuscript. LA and ES compiled and manipulated the data. LA, ES, LS, and OS contributed to the literature review, to conceptualization and writing sections of the manuscript as well as to interpreting the findings. All authors contributed to the article and approved the submitted version.

## Funding

This study was performed under the “ERA.Net RUS plus” program (LifeTraR), funded by the Estonian Research Agency [project number MOBERA24] under the project no. PRG71; and partially by the Academy of Finland Love Age project (no. 317808) and the NetResilience consortium funded by the Strategic Research Council within the Academy of Finland (grant no. 345183). This manuscript uses data from SHARE Waves 1, 2, 3, 4, 5, 6, 7, and 8 (DOIs: 10.6103/SHARE.w1.710, 10.6103/SHARE.w2.710, 10.6103/SHARE.w3.710, 10.6103/SHARE.w4.710, 10.6103/SHARE.w5.710, 10.6103/SHARE.w6.710, 10.6103/SHARE.w7.711, 10.6103/SHARE.w8.100, 10.6103/SHARE.w8ca.100), see (52) for methodological details. The SHARE data collection has been funded by the European Commission, DG RTD through FP5 (QLK6-CT-2001-00360), FP6 (SHARE-I3: RII-CT-2006-062193, COMPARE: CIT5-CT-2005-028857, SHARELIFE: CIT4-CT-2006-028812), FP7 (SHARE-PREP: GA N°211909, SHARE-LEAP: GA N°227822, SHARE M4: GA N°261982, DASISH: GA N°283646), by Horizon 2020 (SHARE-DEV3: GA N°676536, SHARE-COHESION: GA N°870628, SERISS: GA N°654221, SSHOC: GA N°823782) and by DG Employment, Social Affairs & Inclusion through *VS* 2015/0195, *VS* 2016/0135, *VS* 2018/0285, *VS* 2019/0332, and *VS* 2020/0313. Additional funding from the German Ministry of Education and Research, the Max Planck Society for the Advancement of Science, and the U.S. National Institute on Aging (U01_AG09740-13S2, P01_AG005842, P01_AG08291, P30_AG12815, R21_AG025169, Y1-AG-4553-01, IAG_BSR06-11, OGHA_04-064, HHSN271201300071C, RAG052527A), as well as from various national funding sources is gratefully acknowledged (see www.share-project.org). This paper uses data from WHO’s Study on Global Ageing and Adult Health (SAGE). SAGE is supported by the U.S. National Institute on Aging through Interagency Agreements (OGHA 04034785; YA1323-08-CN-0020; Y1-AG-1005-0) and through the research grants R01-AG034479 and R21-AG034263.

## Conflict of interest

The authors declare that the research was conducted in the absence of any commercial or financial relationships that could be construed as a potential conflict of interest.

## Publisher’s note

All claims expressed in this article are solely those of the authors and do not necessarily represent those of their affiliated organizations, or those of the publisher, the editors and the reviewers. Any product that may be evaluated in this article, or claim that may be made by its manufacturer, is not guaranteed or endorsed by the publisher.

## Footnotes

^1^Previously, we conducted an analysis of the 50+ population living in both urban and rural areas, and the results indicated that Russians in Estonia living in rural areas are at highest risk of cognitive impairment. However, the sample of Russians in rural Estonia was small (n = 121), and the confidence interval bounds were large.

^2^First- and second-generation Russians in Estonia were distinguished based on country of birth; for the second generation, we included those who were born in Estonia and whose parents were born outside Estonia. Second-generation Russian women indicated significantly higher odds of immediate recall impairment than Estonian women, and significantly higher odds of fluency impairment than all other groups, despite being on average much younger than the rest. However, the sample size for this group (*n* = 238), including those impaired (*n* = 15), was too small to make reliable conclusions.

^3^We also ran models distinguishing age at migration, creating the following groups: Estonians in Estonia, Russians born in Estonia (i.e., second generation), Russians who migrated to Estonia before age 18 (i.e., child migrants), Russians who migrated to Estonia between ages 18–24 (usually considered the most active migration age), Russians who migrated at age 25 or later, and Russians in Russia. These groups differ in their age structure with Russians born in Estonia being on average the youngest, and those migrating at age 25 or later being on average the oldest. The mean age of those who migrated between ages 18–24 was similar to Estonians in the study, while the mean age of child migrants was similar to Russians in Russia (Supplementary Figure S1). Fluency impairment odds were the highest for Russian women born in Estonia, and these became significantly different from those migrating at age 25 or older after controlling for age, remaining so throughout the models. Regression outcomes comparing groups without those born in Estonia indicated no significant differences in recall impairment neither in fluency between the groups among both men and women (Supplementary Table S7).
