# Supplementary material for: Comparing the cognitive functioning of middle-aged and older foreign-origin population in Estonia to host and origin populations
Source: Front Public Health. 2023 Jul 12;11:1058578. doi: 10.3389/fpubh.2023.1058578 (PMC10382126; doi:10.3389/fpubh.2023.1058578)
Supplement: Supplementary file 1 [file Data_Sheet_1.docx]

**Appendix 1**

**Table 5. Construction of variables in the analysis based on questions from SAGE and SHARE**

| **Topic** | **SHARE** | **Response options** | **SAGE** | **Response options** | **Variable in analysis** |
| --- | --- | --- | --- | --- | --- |
| **Financial situation** | Thinking of your household's total monthly income, would you say that your household is able to make ends meet? | 1. With great difficulty, 2. With some difficulty, 3. Fairly easily, 4. Easily | Would you say your household's financial situation is…? | Very good / Good / Moderate / Bad / Very bad | 1. Have difficulty (incl SHARE: "With great difficulty" and SAGE: "Bad/ Very bad"); 0. OK |
| **Smoking** | Do you smoke at the present time? | Yes/ No | Do you currently use (smoke, sniff or chew) any tobacco products such as cigarettes, cigars, pipes, chewing tobacco or snuff? | Yes/ No | 1. Current smoker; 0. Not a current smoker |
| **Alcohol drinking** | During the last 3 months, how often did you drink any alcoholic beverages, like beer, cider, wine, spirits or cocktails? | Daily or almost every day, Five or six days a week, Three or four days a week, Once or twice a week  Once or twice a month, Less than once a month,  Not at all in the last 3 months | In the last 12 months, how frequently [on how many days] on average have you had at least one alcoholic drink? | No days,  < 1 per month , 1-3 days per month,   1-4 days per week, 5+ days per week | 1. Never (incl. SAGE: "No Days"; SHARE "Not at all…"); 2. Sometimes (SAGE: "< 1 per month", "1-3 days per month"; SHARE: "Once or twice a month", "Less than once a month"), 3. Often (SAGE/ SHARE: 1+ a week) |
| **Depression** | In the last month, have you been sad or depressed? | Yes/ No | During the last 12 months, have you had a period lasting several days when you felt sad, empty or depressed? | Yes/ No | 1. Depressive caseness (SAGE: Yes for all three questions; SHARE: Yes for three issues), 0. No depressiveness |
|  | What are your hopes for the future? | 1. Any hopes mentioned 2. No hopes mentioned | During the last 12 months, have you had a period lasting several days when you lost interest in most things you usually enjoy such as personal relationships, work or hobbies/recreation? | Yes/ No |  |
|  | In the last month, have you felt that you would rather be dead? | 1. Any mention of suicidal feelings or wishing to be dead 2. No such feelings | During the last 12 months, have you had a period lasting several days when you have been feeling your energy decreased or that you are tired all the time? | Yes/ No |  |
|  | Do you tend to blame yourself or feel guilty about anything? | 1. Obvious excessive guilt or self-blame 2. No such feelings 3. Mentions guilt or self-blame, but it is unclear if these constitute obvious or excessive guilt or self-blame |  |  |  |
|  | Have you had trouble sleeping recently? | 1. Trouble with sleep or recent change in pattern 2. No trouble sleeping |  |  |  |
|  | In the last month, what is your interest in things? | 1. Less interest than usual mentioned 2. No mention of loss of interest 3. Non-specific or uncodeable response |  |  |  |
|  | Have you been irritable recently? | Yes/ No |  |  |  |
|  | What has your appetite been like? | 1. Diminution in desire for food 2. No diminution in desire for food 3. Non-specific or uncodeable response |  |  |  |
|  | In the last month, have you had too little energy to do the things you wanted to do? | Yes/ No |  |  |  |
|  | How is your concentration? For example, can you concentrate on a television programme, film or radio programme? | 1. Difficulty in concentrating on entertainment 2. No such difficulty mentioned |  |  |  |
|  | What have you enjoyed doing recently? | 1. Fails to mention any enjoyable activity 2. Mentions ANY enjoyment from activity |  |  |  |
|  | In the last month, have you cried at all? | Yes/ No |  |  |  |
| **Trust** | Generally speaking, would you say that most people can be trusted or that you can't be too careful in dealing with people? Not looking at card 35 anymore, please tell me on a scale from 0 to 10, where 0 means you can't be too careful, and 10 means that most people can be trusted. | Scale 0 … 10 | Generally speaking, would you say that most people can be trusted, or that you can't be too careful in dealing with people? | 1. Can be trusted 2. Can't be too careful | 1. Low/ No trust (SAGE: 2; SHARE: 0…4), 2. Trust in people (SAGE: 1; SHARE: 5...10) |
| **Satisfaction with social relations** | Overall, how satisfied are you with the relationships we have just talked about? Please answer on a scale from 0 to 10, where 0 means completely dissatisfied, and 10 means completely satisfied. | Scale 0 … 10 | How satisfied are you with your personal relationships? | 1. Very satisfied, 2. Satisfied, 3. Neither satisfied nor dissatisfied, 4. Dissatisfied, 5. Very dissatisfied | 1. Dissatisfied (SAGE: 4/5; SHARE: 0/4), 2. Neutral (SAGE: 3; SHARE: 5/6), 3. Satisfied (SAGE: 1/2; SHARE: 7/10) |
| **Receipt of care** | Thinking about the last twelve months, has any family member from outside the household, any friend or neighbour given you [or/or/or/or] [your/your/your/your] [husband/wife/partner/partner] personal care or practical household help? | Yes/ No | In the last 12 months, has anyone in the household received any financial or in-kind support from your family (children, siblings or parents) and relatives (other kin) who do not live with you? | Yes/ No | 1. Has received care (Yes on any of the questions), 0. Has not received care (No on any of the questions) |
|  | Is there any other family member from outside the household, friend or neighbour who has given you [or/or /or/or] [your/your/your/your] [husband/wife/partner/partner] personal care or practical household help? | Yes/ No |  |  |  |
|  | And is there someone living in this household who has helped you regularly during the last twelve months with personal care, such as washing, getting out of bed, or dressing?  IWER: By regularly, we mean daily or almost daily during at least three months. We do not want to capture help during short-term sickness. | Yes/ No |  |  |  |

**Appendix 2 – Cognitive Functioning Instruments**

1. **Cognitive functioning instruments in the SAGE Russia 2007-2010 survey [42, 50]**
   1. **Verbal fluency**

Q: Now we are going to ask you to think of animals and name as many as you can. I am going to give you one minute and I want to see how many animals you can name.

INTERVIEWER: See Interviewers Manual instructions about what is acceptable and what is not. If respondent stops before the end of the minute, encourage them to try to name more animals. If there is a silence of about 15 seconds, prompt them to continue or repeat the basic instructions.

Q236: Total score (number of animals named correctly).

- 1. **Verbal recall**

Q: We are now going to test your memory. I know these questions may be difficult to answer, but please try to provide an answer. I am going to read you a list of words. Listen to them carefully and try to remember as many of them as you can, not necessarily in order. I will ask you to repeat them again after some time.

INTERVIEWER: you can use the table below to assist you with scoring.

List of words in Russian (and English translation): Рука (arm), Кровать (bed), Самолет (plane), Собака (dog), Часы (clock), Велосипед (bike), Ухо (ear), Молоток (hammer), Стул (chair), Кошка (cat).

*According to the Russian national report (WHO 2014), the respondent was asked to name the list again after about 10 minutes*.

Q: 2 trial: I will read the list to you again, and then again when I am done, repeat them after me.

Q: 3 trial: One final time - I will read the list and when I am done, you repeat as many as you can remember.

Q2525/ Q2528/ Q2531: Number of words recalled correctly at Trial x – *we used this indicator (Q2525) for immediate verbal recall*.

1. **Cognitive functioning instruments in the SHARE Estonia 2010-2011 survey [51]**
   1. **Verbal fluency**

CF009: Now I would like you to name as many different animals as you can think of. You have one minute to do this. Ready, go.

IWER: Allow one minute precisely. If the subject stops before the end of the time, encourage them to try to find more words. If they are silent for 15 seconds repeat the basic instruction ('I want you to tell me all the animals you can think of'). No extension on the time limit is made in the event that the instruction has to be repeated.

- 1. **Verbal recall**

CF007: Now, I am going to read a list of words from my computer screen. We have purposely made the list long so it will be difficult for anyone to recall all the words. Most people recall just a few. Please listen carefully, as the set of words cannot be repeated. When I have finished, I will ask you to recall aloud as many of the words as you can, in any order. Is this clear?

*There were four different word lists, in Estonian and in Russian, that the computer could generate (i.e. not all people received the same list to repeat), and the interviewer could read out:*

*Lists in Estonian (English translation provided in the brackets by the article authors):*

**Nimekiri nr 1 Nimekiri nr 2 Nimekiri nr 3 Nimekiri nr 4**
Hotell (hotel) Taevas (sky) Naine (woman) Vesi (water)
Jõgi (river) Laht (bay) Kalju (cliff) Kirik (church)
Puu (tree) Lipp (flag) Veri (blood) Arst (doctor)
Nahk (skin) Dollar (dollar) Nurk (corner) Loss (castle)
Kuld (gold) Ema (mother) Kingad (shoes) Tuli (fire)
Turg (market) Masin (machine) Kiri (letter) Aed (garden)
Paber (paper) Kodu (home) Tüdruk (girl) Meri (sea)
Laps (child Kera (ball) Maja (house) Küla (village)
Keiser (emperor) Kool (shool) Org (valley) Poiss (boy)
Raamat (book) Või (butter) Mootor (motor) Laud (table)

*Lists in Russian (English translation provided in the brackets by the article authors):*

**Список No 1 Список No 2 Список No 3 Список No 4**Номер (number) Небо (sky) Сестра (sister) Вода (water)
Река (river) Океан (ocean) Скала (cliff) Собор (church)
Дерево (tree) Флаг (flag) Кровь (blood) Врач (doctor)
Кожа (skin) Доллар (dollar) Угол (corner) Крепость (castle)
Золото (gold) Жена (wife) Туфли (shoes) Огонь (fire)
Рынок (market) Машина (machine) Письмо (letter) Сад (garden)
Бумага (paper) Изба (monastery) Девочка (girl) Море (sea)
Ребенок (child) Земля (earth) Здание (building) Село (village)
Король (king) Школа (school) Долина (valley) Мальчик (boy)
Книга (book) Масло (butter) Мотор (motor) Стол (table)

*After asking other questions, in about 10 minutes:*

CF113-CF116: A little while ago, I read you a list of words and you repeated the ones you could remember. Please tell me any of the words that you can remember now?

IWER: Write words on sheet provided. Allow up to one minute for recall. Enter the words respondent correctly recalls.

**Appendix 3 – Robustness Checks Using Differently Defined Study Groups**

**Table 6. Number and proportion of cognitively impaired people by sex and groups defined according to place of birth and age at migration**

|  | **Fluency** | | **Immediate recall** | |
| --- | --- | --- | --- | --- |
|  | **Men** | **Women** | **Men** | **Women** |
| **Estonians (no. impaired)** | 202 | 295 | 157 | 160 |
| **% impaired** | 21,6 | 20,6 | 16,8 | 11,2 |
| **Russians born in Estonia (no. impaired)** | 16 | 32 | 15 | 17 |
| **% impaired** | 17,4 | 21,9 | 16,3 | 11,6 |
| **Russians migrated before age 18 (no. impaired)** | 30 | 42 | 29 | 40 |
| **% impaired** | 23,8 | 20,6 | 23 | 19,6 |
| **Russians migrated between age 18-24 (no. impaired)** | 39 | 57 | 45 | 60 |
| **% impaired** | 22,8 | 21,4 | 26,3 | 22,5 |
| **Russians migrated at age 25 or later (no. impaired)** | 35 | 60 | 40 | 55 |
| **% impaired** | 24,8 | 26,6 | 28,4 | 24,3 |
| **Russians in Russia (no. impaired)** | 181 | 376 | 107 | 214 |
| **% impaired** | 23,8 | 23,8 | 14,1 | 13,6 |
|  |  |  |  |  |
| **Pearson Chi2 (group differences)** | 0,688 | 0,203 | 0,000 | 0,000 |

**Table 7. Coefficients of cognitive impairment from finally adjusted binary logistic regression models for different population groups aged 50+, SHARE Estonia 2010- 2011 and SAGE Russia 2007-2010.**

|  | **Groups without those born in Estonia** | | | | |  | **Groups by age at migration (six groups)** | | | | |  | **Groups by age at migration (five groups; without those born in Estonia)** | | | | |
| --- | --- | --- | --- | --- | --- | --- | --- | --- | --- | --- | --- | --- | --- | --- | --- | --- | --- |
|  | **Immediate recall** | |  | **Fluency** | |  | **Immediate recall** | |  | **Fluency** | |  | **Immediate recall** | |  | **Fluency** | |
|  | **Men** | **Women** |  | **Men** | **Women** |  | **Men** | **Women** |  | **Men** | **Women** |  | **Men** | **Women** |  | **Men** | **Women** |
|  | **OR (95 % CI)** | **OR (95 % CI)** |  | **OR (95 % CI)** | **OR (95 % CI)** |  | **OR (95 % CI)** | **OR (95 % CI)** |  | **OR (95 % CI)** | **OR (95 % CI)** |  | **OR (95 % CI)** | **OR (95 % CI)** |  | **OR (95 % CI)** | **OR (95 % CI)** |
| **Estonians** | 0.662 (0.478 - 0.916) | 0.619 (0.460 - 0.833) |  | 0.912 (0.668 - 1.246) | 1.179 (0.907 - 1.533) |  | 0.723 (0.449 - 1.165) | 0.704 (0.467 - 1.062) |  | 0.996 (0.623 - 1.592) | 1.136 (0.782 - 1.651) |  | 0.700 (0.434 - 1.128) | 0.699 (0.464 - 1.054) |  | 0.993 (0.621 - 1.590) | 1.129 (0.777 - 1.640) |
| **Russians in Russia** | 0.783 (0.513 - 1.194) | 0.800 (0.570 - 1.123) |  | 0.953 (0.653 - 1.391) | 1.162 (0.862 - 1.566) |  | 1.127 (0.494 - 2.567) | 1.331 (0.663 - 2.675) |  | 1.043 (0.624 - 1.744) | 1.121 (0.750 - 1.676) |  | 0.827 (0.479 - 1.427) | 0.905 (0.579 - 1.414) |  | 1.039 (0.620 - 1.741) | 1.112 (0.743 - 1.663) |
| **Russians in Estonia (all)** | 1 | 1 |  | 1 | 1 |  | - | - |  | - | - |  | - | - |  | - | - |
| **Russians born in Estonia** | - | - |  | - | - |  | 1.040 (0.544 - 1.987) | 1.182 (0.695 - 2.011) |  | 1.048 (0.486 - 2.258) | 1.944 (1.104 - 3.425) |  | - | - |  | - | - |
| **Migrated before age 18** | - | - |  | - | - |  | 1.142 (0.642 - 2.031) | 1.208 (0.740 - 1.971) |  | 1.229 (0.657 - 2.300) | 0.972 (0.589 - 1.606) |  | 1.030 (0.540 - 1.977) | 1.189 (0.700 - 2.019) |  | 1.223 (0.653 - 2.288) | 0.976 (0.591 - 1.611) |
| **Migrated between age 18 -24** | - | - |  | - | - |  | 0.836 (0.485 - 1.440) | 0.929 (0.595 - 1.452) |  | 1.077 (0.605 - 1.920) | 0.909 (0.568 - 1.452) |  | 1.129 (0.635 - 2.007) | 1.223 (0.750 - 1.993) |  | 1.071 (0.601 - 1.910) | 0.903 (0.565 - 1.442) |
| **Migrated at age 25 or later** | - | - |  | - | - |  | 1 | 1 |  | 1 | 1 |  | 1 | 1 |  | 1 | 1 |
|  |  |  |  |  |  |  |  |  |  |  |  |  |  |  |  |  |  |
| **R^2^** | 0.1640 | 0.1947 |  | 0.1364 | 0.1508 |  | 0.1641 | 0.1973 |  | 0.1362 | 0.1532 |  | 0.1641 | 0.1949 |  | 0.1366 | 0.1509 |
| **N** | 2011 | 3570 |  | 2011 | 3570 |  | 2096 | 3712 |  | 2096 | 3712 |  | 2011 | 3570 |  | 2011 | 3570 |
