# Supplementary figures and images for: Comparing the cognitive functioning of middle-aged and older foreign-origin population in Estonia to host and origin populations
Source: Front Public Health. 2023 Jul 12;11:1058578. doi: 10.3389/fpubh.2023.1058578 (PMC10382126; doi:10.3389/fpubh.2023.1058578)

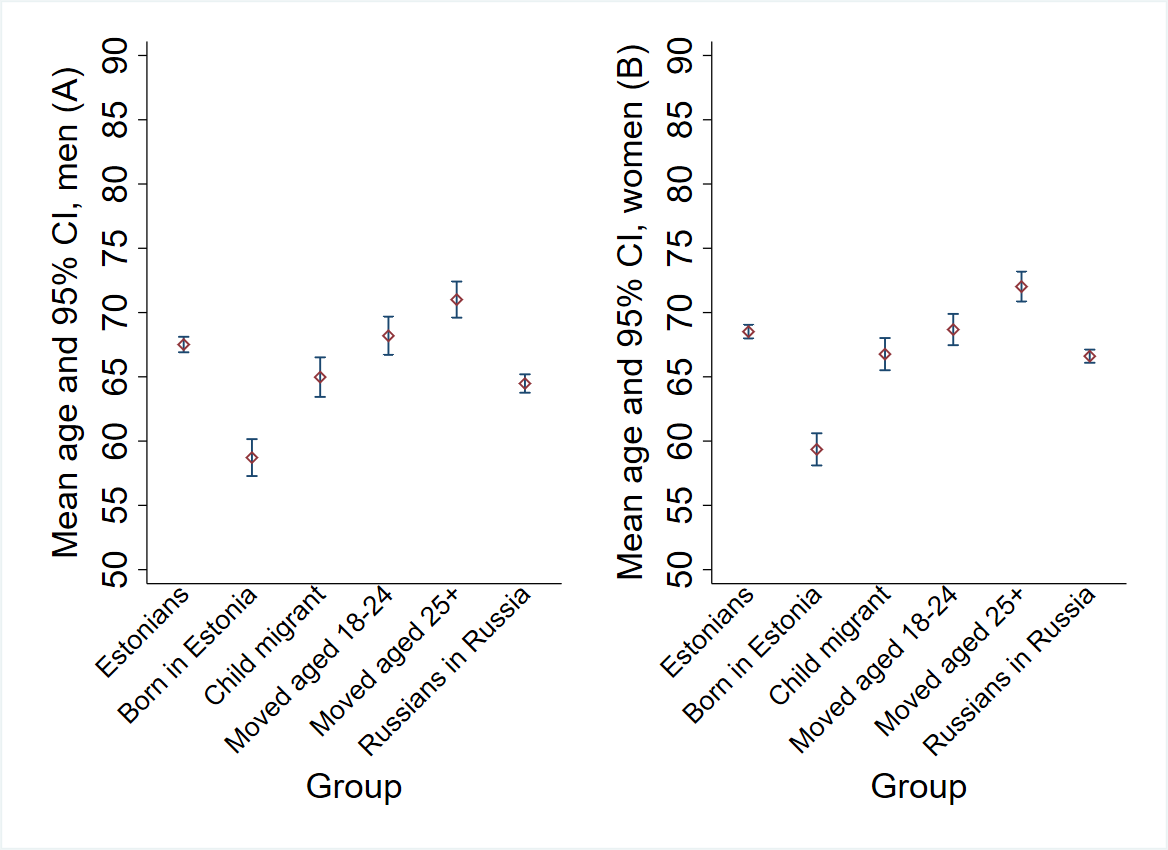

Supplement: Supplementary file 2 [file Figure_1.TIF]
